# Zinc limitation in *Klebsiella pneumoniae* profiled by quantitative proteomics influences transcriptional regulation and cation transporter-associated capsule production

**DOI:** 10.1186/s12866-021-02091-8

**Published:** 2021-02-10

**Authors:** A. Sukumaran, S. Pladwig, J. Geddes-McAlister

**Affiliations:** Department of Molecular and Cellular Biology, University of Guelph, Guelph, Ontario N1G 2W1 Canada

**Keywords:** *Klebsiella pneumoniae*, Zinc limitation, Cation transporter, Polysaccharide capsule, Quantitative proteomics, Secretome

## Abstract

**Background:**

Microbial organisms encounter a variety of environmental conditions, including changes to metal ion availability. Metal ions play an important role in many biological processes for growth and survival. As such, microbes alter their cellular protein levels and secretion patterns in adaptation to a changing environment. This study focuses on *Klebsiella pneumoniae*, an opportunistic bacterium responsible for nosocomial infections. By using *K. pneumoniae*, we aim to determine how a nutrient-limited environment (e.g., zinc depletion) modulates the cellular proteome and secretome of the bacterium. By testing virulence in vitro*,* we provide novel insight into bacterial responses to limited environments in the presence of the host.

**Results:**

Analysis of intra- and extracellular changes identified 2380 proteins from the total cellular proteome (cell pellet) and 246 secreted proteins (supernatant). Specifically, HutC, a repressor of the histidine utilization operon, showed significantly increased abundance under zinc-replete conditions, which coincided with an expected reduction in expression of genes within the *hut* operon from our validating qRT-PCR analysis. Additionally, we characterized a putative cation transport regulator, ChaB that showed significantly higher abundance under zinc-replete vs. -limited conditions, suggesting a role in metal ion homeostasis. Phenotypic analysis of a *chaB* deletion strain demonstrated a reduction in capsule production, zinc-dependent growth and ion utilization, and reduced virulence when compared to the wild-type strain.

**Conclusions:**

This is first study to comprehensively profile the impact of zinc availability on the proteome and secretome of *K. pneumoniae* and uncover a novel connection between zinc transport and capsule production in the bacterial system.

**Supplementary Information:**

The online version contains supplementary material available at 10.1186/s12866-021-02091-8.

## Background

*Klebsiella pneumoniae* is an opportunistic, Gram-negative bacterium that primarily causes nosocomial infections. It is found ubiquitously in the environment, commonly in soil and surface water, but is also very prevalent in medical settings [1]. *K. pneumoniae* is primarily acquired in a hospital setting via person-to-person contact or through hospital equipment (e.g., catheters or ventilators), resulting in pneumonia and urinary tract infections but can lead to liver abscesses or soft tissue infections in more serious cases [2]. The prevalence of antimicrobial resistance and the emergence of new strains underscore the substantial threat of *K. pneumoniae* to immunocompromised individuals [3, 4]. Moreover, such factors drive the need to define mechanisms of virulence, environmental adaptation strategies, and uncover new therapeutic options [5].

Transition metals play a crucial role in numerous functions within all living organisms. Their acquisition is essential to microbial survival, but similarly, the maintenance of intracellular concentration is of equal importance. Elevated intracellular levels of metals have negative effects on microbial fitness [6]. The most common transition metals relevant to microbe fitness include redox (e.g., iron or copper) and non-redox (e.g., zinc) metals. Previous studies investigating the relationship between *K. pneumoniae* and transition metal availability focused primarily on iron limitation. These studies found an iron-associated impact on bacterial growth, capsule development, and protease secretion [7–9]. Moreover, *K. pneumoniae* produces siderophores, iron-chelating molecules that can acquire free iron from the environment or sequester it from host proteins, to regulate intracellular iron levels during infection [10–12].

Next to iron, zinc is the second most abundant transition metal [13]. As a non-redox metal, zinc is stable and can serve as an electrophilic catalyst, which allows it to either participate in catalysis or maintain stability [14]. In bacteria, zinc is essential for enzyme activity, DNA replication and repair, and regulation of transcription factors [15]. The maintenance of zinc levels is primarily controlled by the zinc uptake regulator (Zur) [16]. Microarray analysis of zinc limitation in *Escherichia coli* identified upregulated genes proposed to interact with Zur to regulate zinc uptake [17]. Zur also interacts with ZitB, a zinc exporter, hinting at a possible role in both sides of zinc homeostasis [18]. Under zinc limited conditions, *Mycobacterium* expresses four zinc-independent alternative ribosomal proteins (AltRP) proposed to interact with the Zur regulon [19]. Analysis of *Mycobacterium smegmatis* identified that these proteins are essential for survival under zinc limited conditions, and mutations to the *altRP* operon affected cell morphology [20]. Conversely, in fungal pathogens, zincosomes have been described and serve to both detoxify excess zinc and mobilize zinc upon deprivation, supporting a role in metal ion homeostasis under replete conditions [21, 22].

The acquisition of zinc is important for microbial survival within the host. For example, during invasion by pathogenic microbes, the host will sequester transition metals from the environment, thereby restricting nutrients from the pathogen - a term coined nutritional immunity [15]. Conversely, elevated intracellular levels also have toxic effects [23]. Alongside limitation of free zinc ions, host immune cells increase intracellular zinc concentrations upon phagocytosis to induce metal  toxicity in response to invasion of *Mycobacterium tuberculosis*, *Streptococcus pyogenes*, and *E. coli* [24–26]. To date, the acquisition and regulation of zinc has been described at the transcriptional level in multiple bacterial species, which provides valuable insight into bacterial adaptability and the relevance of zinc within the host. However, investigation of the impact of zinc limitation in *K. pneumoniae* (a clinically-relevant bacterium) and defining the influence of zinc at the protein-level, which provides novel insight into protein production in response to environmental stresses, has yet to be explored. Information on protein production, abundances, and pathway involvement can contribute to our mechanistic understanding of bacterial adaptation from a systems perspective [27].

The present study aims to define how *K. pneumoniae* responds to a host-like setting (i.e., nutrient limitation) by characterizing the relationship between zinc availability and intra- and extracellular bacterial processes via quantitative proteomics*.* Using mass spectrometry-based proteomics, we identified differentially abundant proteins under zinc-limited and -replete conditions in the cellular proteome (cell pellet) and secretome (culture supernatant) of *K. pneumoniae*. We validated our proteomic results by quantifying the impact of the identified histidine utilization repressor (HutC) on its target genes at the transcriptional level. We identified an under characterized protein, ChaB, that increased significantly in abundance under zinc-replete conditions. In silico analysis putatively identified ChaB as a cation transport regulator. Deletion of *chaB* resulted in *K. pneumoniae* zinc-dependent growth, differences in ion utilization, and a reduced capsule production compared to wild-type (WT). Virulence was tested by replicating an infection setting using an in vitro assay to measure host cell death, whereupon, bacterial culturing in minimal compared to rich media resulted in impaired virulence of the deletion strain compared to WT [28]. Overall, by mimicking an infection setting in the host through limitation of metal ion availability, this study provides new insight into the global impact of zinc availability on *K. pneumoniae.*

## Results

### Profiling of the *K. pneumoniae* cellular proteome influenced by zinc availability impacts transcriptional and transport processes

**Fig. 1:**
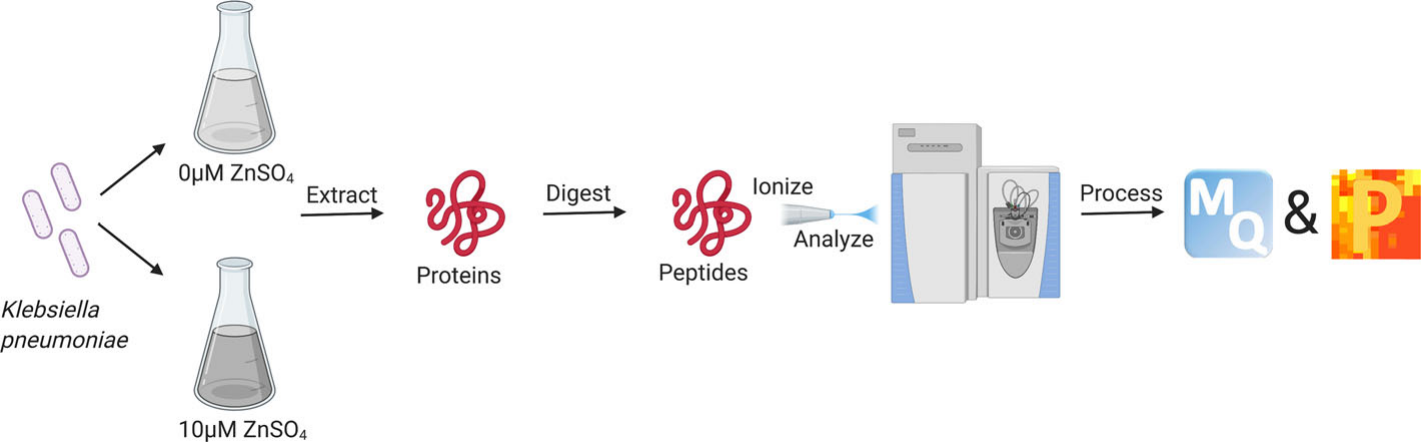
Mass spectrometry-based proteomics workflow of *K. pneumoniae*. Bottom-up proteomics workflow for profiling the cellular proteome and secretome of *K. pneumoniae* under zinc-limited (M9 minimal media with 0 μM zinc) and zinc replete (M9 minimal media supplemented with 10 μM zinc) conditions. Proteins were extracted from cells and supernatant followed by enzymatic digestion and purification on C18 resin tips. The purified peptides are measured in the first MS scan and peptide fragmentation patterns are observed in the second MS scan (MS/MS). The data is processed and analyzed using the publicly available MaxQuant and Perseus platforms. Figure generated with Biorender.com

**Fig. 2:**
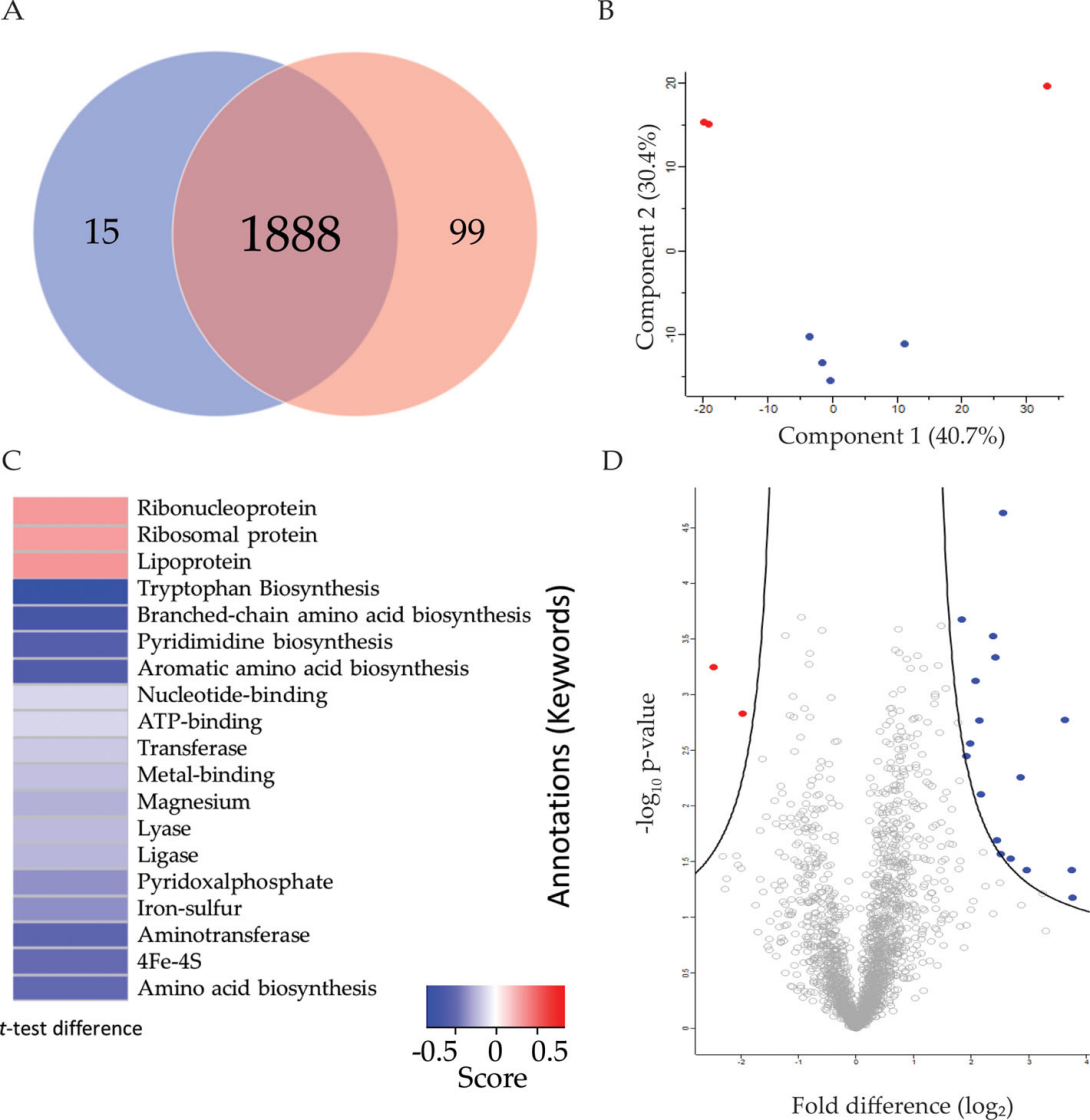
Cellular proteome profiling of *K. pneumoniae* cultured in zinc-limited and -replete media. **a** Venn diagram depicting total unique proteins identified in limited (red, 15) and replete (blue, 99) conditions. **b** Principal component analysis. Variance amongst biological replicates of cells grown in limited (red, top) and replete (blue, bottom) conditions was separated based on component 1 (40.7%) and component 2 (30.4%). **c** 1D-annotation enrichment. UniProt keywords enriched in the replete condition relative to the limited. Statistical significance confirmed by Student’s *t-*test (*p-value* ≤ 0.05, FDR = 0.05, score < − 0.5, < 0.5). **d** Volcano plot depicting proteins present in cellular proteome. Proteins in blue (right) are significantly abundant under replete conditions. Proteins in red (left) are significantly abundant under limited conditions. Statistical analysis was performed using a Student’s *t*-test (*p-value* ≤ 0.05; FDR = 0.05; S0 = 1). Experiment performed in biological quadruplicate and technical duplicate

Under infection conditions, the host will sequester free zinc ions to deprive the invading pathogen of this vital micronutrient [29]. Free zinc concentration in the serum is expected in the micromolar range, in agreement with a recent study in *E. coli* [30]. Here, we evaluate the impact of zinc availability by quantitative proteomic profiling of *K. pneumoniae* WT cells grown in zinc-limited (0 μM) and -replete (10 μM) media (Fig. 1). We identified a total of 2380 proteins (representing 46.4% of encoding regions) in the cellular proteome. Upon filtering for proteins identified in three of four biological replicates, we further analyzed 2002 proteins. Notably, we identified proteins unique to each growth condition: 15 proteins in limited and 99 proteins in replete (Fig. 2a). A principal component analysis (PCA) defined separation of conditions (limited vs. replete media) (component 2, 30.4%) and separation of biological factors (component 1, 40.7%) (Fig. 2b). Replicate reproducibility was 92.7% amongst the limited biological replicates and 96.7% amongst the replete biological replicates.

Using a 1D-annotation enrichment analysis, which demonstrates a global overview of changes to protein abundance by testing every annotation term if the corresponding numerical values have a preference to be systematically larger or smaller than the global distribution of the values for all proteins, we aimed to identify categories of proteins based on Uniprot Keywords that were enriched or de-enriched under limited or replete conditions [31]. We identified three categories enriched under replete conditions, including proteins associated with lipoproteins and ribosomal-associated proteins (Fig. 2c). Conversely, we observed an enrichment of 4Fe-4S, iron-sulfur, metal-binding and transferase under limited conditions. These correlate to a reduction in metal ion acquisition, an expected result when analysing the limited conditions where zinc would be less bioavailable.

Next, to define proteins with significant changes in abundance between zinc-limited vs. -replete conditions in the cellular proteome, we performed false discovery rate (FDR)-corrected Student’s *t*-test. We identified 19 proteins whose abundance was significantly different between replete and limited conditions: 17 proteins significantly increased in abundance in replete media vs. two proteins significantly increased in abundance in limited media (Fig. 2d; Table 1). Under replete conditions, the most differentially abundant protein (> 13-fold change) was the histidine utilization repressor (HutC), responsible for regulating the *hut* operon, which is involved in degradation of histidine to glutamate and ammonia [32]. Other proteins highly abundant under replete conditions include phage shock proteins (PspB and PspC) and a thiosulfate transport system permease T protein (CysU), hinting at induction of a stress response to high zinc availability. We also observed a significant change in abundance (> 5-fold change) of an uncharacterized putative cation transporter (ChaB). Conversely, the two proteins significantly increased under limited conditions, include acetolactate synthase small subunit (IlvN) and a polycystic kidney and hepatic disease-type hydroxylase (KPN78578_12210). Taken together, this data demonstrates cellular remodelling under altered zinc conditions and uncovers significantly higher production of a putative cation transporter (ChaB) in replete medium to potentially maintain ion homeostasis.

**Table 1:** Significantly different proteins identified in the cellular proteome of *K. pneumoniae* relative to zinc replete conditions

Protein IDs	Fold difference	Gene names	Protein names
A6TFX2	−5.522	*ilvN*	Acetolactate synthase small subunit
A6T7W1	−3.923	*KPN78578_12210*	PKHD-type hydroxylase
A6THZ7	3.587	*deoA*	Thymidine phosphorylase
A6TGG0	3.783	*ilvY*	Positive regulator for *ilvC*
A6TFL0	3.945	*yibQ*	Uncharacterized protein
A6T709	4.210	*KPN78578_09190*	UPF0434 protein
A6T7L3	4.406	*yciE*	Uncharacterized protein
A6TBM1	4.507	*yohC*	Putative transport protein
A6THH0	5.221	*treR*	Trehalose repressor
A6T810	5.363	*pspB*	Phage shock protein B
A6TAN3	5.442	*chaB*	Cation transport regulator
A6T811	5.691	*pspC*	Phage shock protein
A6TI69	5.862	*KPN_pKPN3p05911*	Uncharacterized protein
A6TC61	6.434	*cysU*	Sulfate, thiosulfate transport system permease T protein
A6T9K1	7.243	*KPN_01841*	Uncharacterized protein
A6TF61	7.816	*yhhA*	Uncharacterized protein
A6TAE9	12.359	*KPN_02142*	Uncharacterized protein
A6T769	13.369	*acyP*	Acylphosphatase
A6T6K9	13.487	*hutC*	Histidine utilization repressor

### Secretome profiling of zinc availability highlights changes in protein secretion patterns

**Fig. 3:**
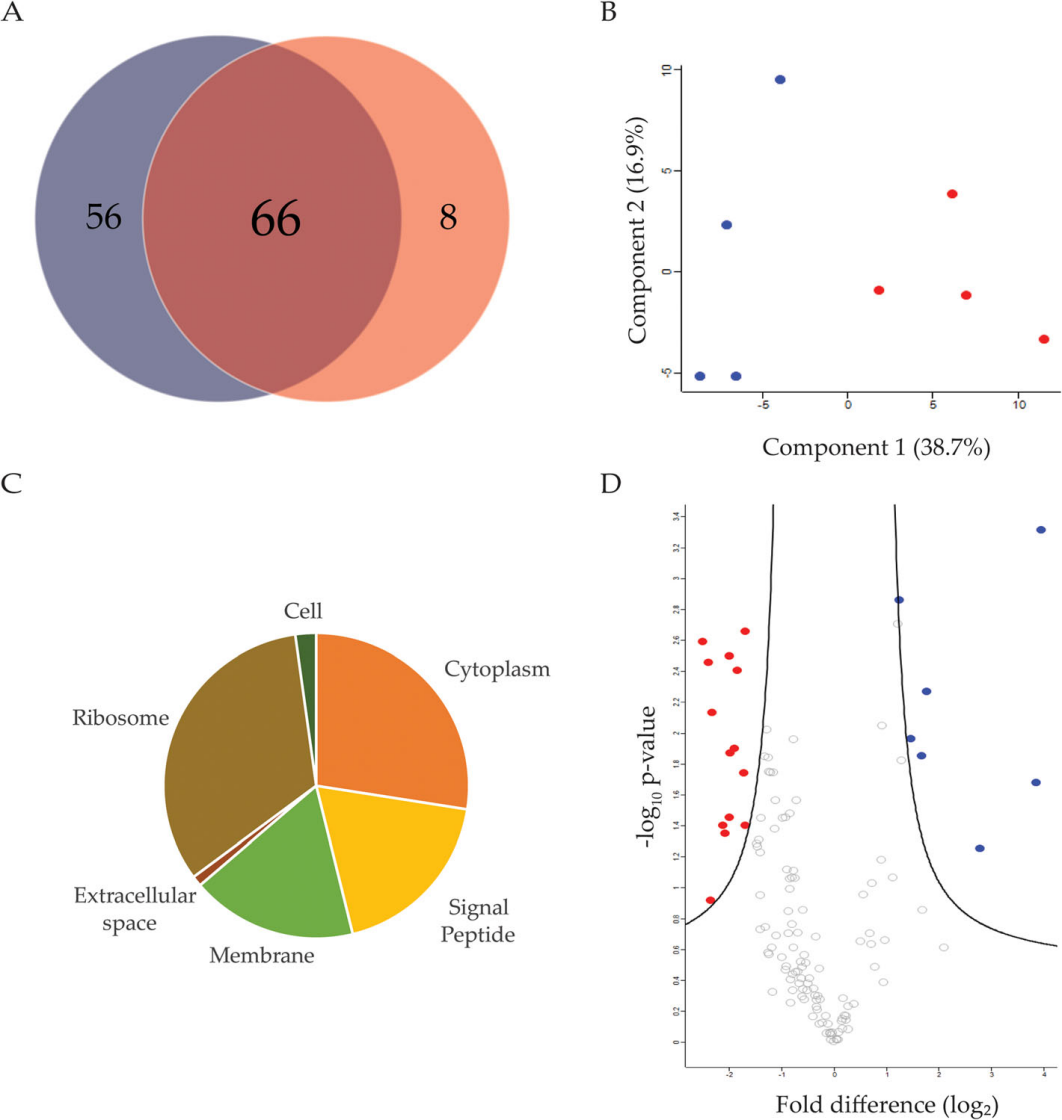
Secretome profiling of *K. pneumoniae* cultured in zinc-limited and -replete media. **a** Venn diagram depicting total proteins identified in limited (red, 56) and replete (blue, 8) conditions. **b** Principal component analysis. Variance amongst biological replicates of cells grown in limited (red, right) and replete (blue, left) conditions separated based on component 1 (38.7%) and component 2 (16.9%). **c** Pie chart depicting Gene Ontology Cellular Compartment categories and presence of signal peptide. **d** Volcano plot depicting proteins present in secretome. Proteins in blue (right) are significantly abundant under replete conditions. Proteins in red (left) are significantly abundant under limited conditions. Statistical analysis was performed using a Student’s *t*-test (*p*-value ≤0.05; FDR = 0.05; S0 = 1). Experiment performed in biological quadruplicate and technical duplicate

Acquisition of zinc from the environment may result in the secretion of proteins associated with sequestering and transport of metal ions into the extracellular space. To identify if *K. pneumoniae* secretes proteins into the extracellular environment under differing zinc concentrations, we performed proteomic profiling of the supernatant (secretome) of *K. pneumoniae* cultures. We identified 246 proteins prior to filtering and performed subsequent analyses on 130 secreted proteins (present in three of four replicates), 56 of which were uniquely present under limited conditions and eight under replete conditions (Fig. 3a). Visualizing the variance of the biological replicates using a PCA plot identified the largest separating component (component 1, 38.7%) distinguishing the limited and replete samples (Fig. 3b). The second largest component (component 2, 16.9%) separated based on biological variance. Amongst the conditions, we found a reproducibility of 80.8 and 78.6% for the limited and replete samples, respectively. Enrichment analysis of the secretome based on Uniprot Keywords did not show category enrichment in either limited or replete conditions. However, analysis of the secreted proteins based on Gene Ontology cellular compartment identified 33 proteins to be associated with conventional secretion patterns (e.g., transmembrane, signal peptide) (Fig. 3c). In addition, we compared our secretome data set to previous proteome profiling of *K. pneumoniae* vesicles and found overlap of 41 (31.5%) protein identifications, supporting our detection of traditional intracellular proteins within the extracellular environment (Supp. Table 2) [33].

Statistical analysis (FDR-corrected Student’s *t*-test) of the secretome dataset identified 21 significantly different proteins, including seven proteins with higher abundance in replete media and 14 proteins that displayed increased abundance in limited media (Fig. 3d; Table 2). Under replete conditions, we identified proteins involved with transport such as a putative periplasmic binding protein (KPN_00624), a taurine transport protein (TauA), and a periplasmic chaperone (HlpA). The most differentially abundant protein (> 15-fold change) under replete conditions was a repressor of methionine biosynthesis, (MetJ). Conversely, 14 proteins displayed an increased abundance under limited conditions, including two chaperones: a heat shock protein (DnaK) and a cold shock protein (CspC), a lipoprotein (MetQ), and an outer membrane protein (OmpX). Proteins displaying the largest fold-difference (> 5-fold change) were two dehydrogenases: dihydrolipoyl dehydrogenase (LpdA) and pyruvate dehydrogenase E1 component (AceE), demonstrating remodeling of the bacterial extracellular environment in response to changing zinc availability.

**Table 2:** Significantly different proteins identified in the secretome of *K. pneumoniae* relative to zinc replete conditions

Protein IDs	Fold difference	Gene name	Protein name
A6T4Q9	-5.672	*lpdA*	Dihydrolipoyl dehydrogenase
A6T4Q7	-5.271	*aceE*	Pyruvate dehydrogenase E1 component
A6T4F8	-5.135	*rpsT*	30S ribosomal protein S20
A6TGT4	-5.043	*pgi*	Glucose-6-phosphate isomerase
A6T6Q8	-4.366	*ompX*	Outer membrane protein X
A6T9L3	-4.238	*adhP*	Alcohol dehydrogenase
A6TG36	-4.018	*atpD*	ATP synthase subunit beta
A6T500	-4.017	*metQ*	Lipoprotein
A6TEW7	-3.970	*rplV*	50S ribosomal protein L22
A6TEW5	-3.743	*rplP*	50S ribosomal protein L16
A6TGQ0	-3.615	*thiE*	Thiamine-phosphate synthase
A6T4F4	-3.306	*dnaK*	Chaperone protein DnaK (HSP70)
A6TEX8	-3.244	*fusA*	Elongation factor G
A6TAZ5	-3.239	*cspC*	Cold shock protein
A6T4Y0	2.361	*hlpA*	Periplasmic molecular chaperone for outer membrane proteins
A6T4W7	2.743	*KPN78578_01770*	UPF0325 protein
A6T653	3.185	*KPN_00624*	Putative periplasmic binding protein/LacI transcriptional regulator
A6T592	3.396	*tauA*	Taurine transport protein
A6TAI1	6.877	*ihfA*	Integration host factor subunit alpha
A6TFP8	14.425	*rpoZ*	DNA-directed RNA polymerase subunit omega
A6TGC7	15.345	*metJ*	Met repressor

### qRT-PCR analysis of *hutC* validates protein-level changes in the presence of zinc and supports a role for zinc in transcriptional repression

**Fig. 4:**
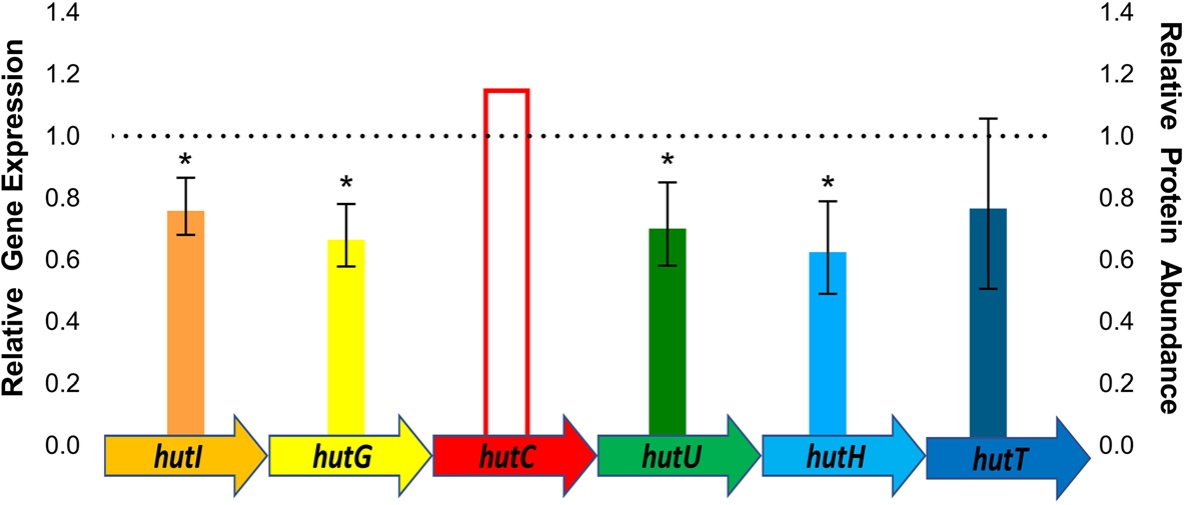
Gene expression analysis of the *hut* operon. Total RNA was extracted from *K. pneumoniae* grown in zinc-limited and -replete media. Values are normalized against the expression of the reference genes, *rho* and *recA*. Values reflect the expression of each gene in the replete condition relative to the limited condition. The asterisk (*) denotes statistically significant expression (one-tail Student’s *t*-test, *p*-value ≤0.05). Data is reflective of three biological replicates and two technical replicates per biological replicate. Ratio of protein abundance of HutC under zinc-replete conditions relative to -limited is denoted by the bar with the red border

Under zinc-replete conditions, we observed a significant increase in abundance of HutC. To validate our proteomic results, we quantified changes in transcript levels of HutC target genes (e.g., *hutG, hutH, hutI, hutT, and hutU*). We performed a gene expression analysis using qRT-PCR on the *hut* operon under limited and replete conditions. Under replete conditions, we report a down-regulation of the *hut* operon genes relative to limited conditions, as expected in the presence of high HutC production (Fig. 4). These results suggest an increase in histidine degradation under zinc limited conditions and support a role for zinc in influencing transcriptional repression in *K. pneumoniae*.

### Characterization of *chaB* and its role in capsule production

**Fig. 5:**
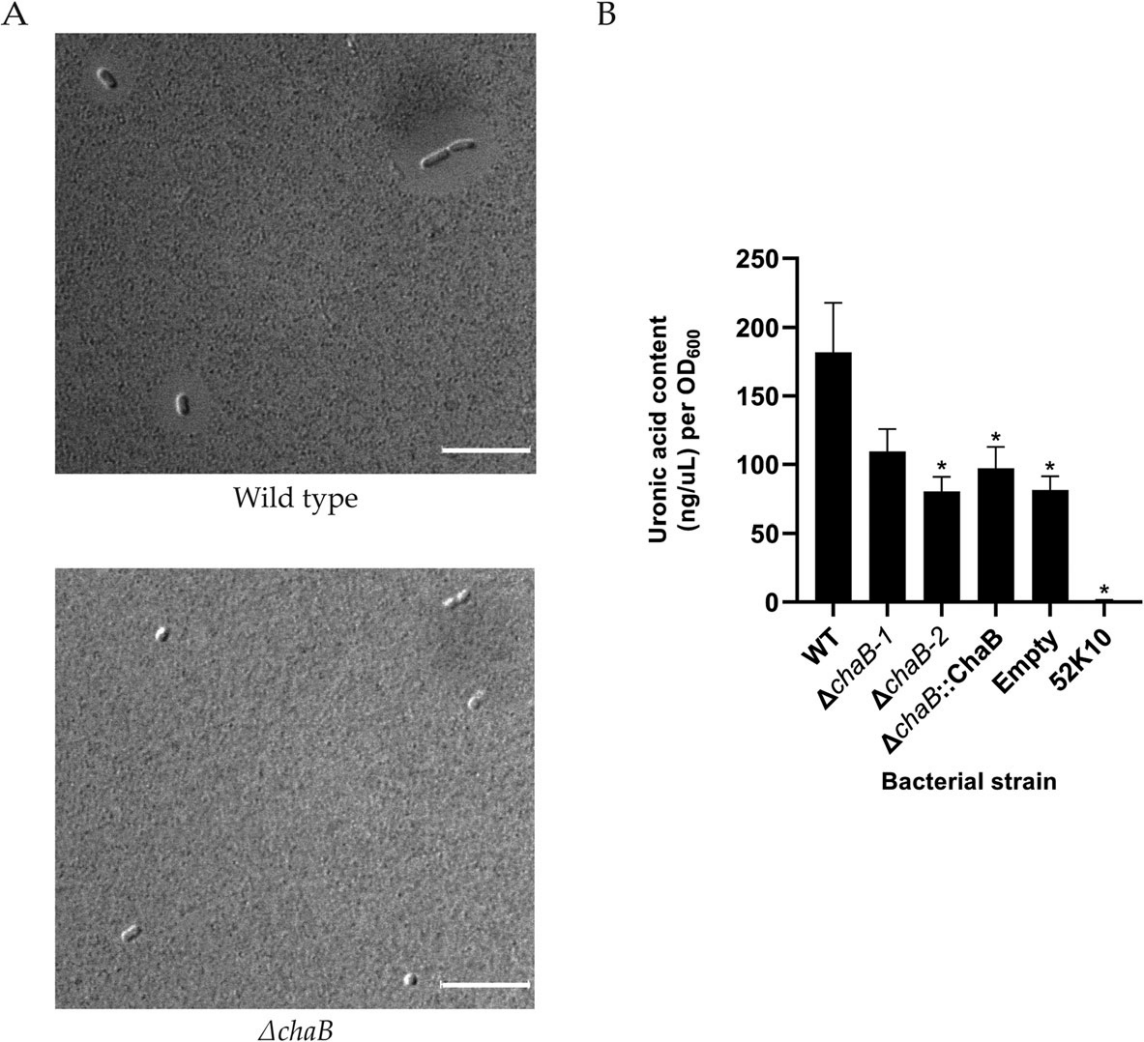
Capsule production of *K. pneumoniae* ∆*chaB.* **a** DIC microscopy at 100x magnification of WT and ∆*chaB* strain at mid-log phase of growth. Scale bar = 100 μm. **b** Uronic acid assay to measure capsule production. Concentration calculated using a glucuronic acid standard curve. *denotes significant difference inferred by ANOVA followed by Tukey HSD test (*p*-value ≤0.05) between WT and tested strains

Under zinc replete conditions, we report the increased abundance (> 5-fold change) of a putative cation transporter, ChaB. To investigate the role of this protein in *K. pneumoniae*, we constructed a deletion strain and evaluated phenotypic differences between ∆*chaB* and WT *K. pneumoniae* . We first performed characterization of ∆*chaB* through visualization of differences in capsule production between the WT and mutant strains. By differential interference contrast (DIC) microscopy, we reported a reduction in capsule size in the ∆*chaB* vs. WT strains (Fig. 5a). To quantify and validate the capsule reduction observed, we performed an uronic acid assay to quantitate the presence of uronic acid as a surrogate for capsule polysaccharide production in the ∆*chaB* strain [34, 35]. We quantified uronic acid present in the WT, ∆*chaB*, ∆*chaB::*ChaB complement, empty vector control, and an acapsular strain (*K. pneumoniae* 52K10). We observed a statistically significant reduction (*p*-value ≤0.05) in uronic acid in the ∆*chaB* strain in comparison to the WT, a similar level of capsule production in the ∆*chaB::*ChaB and empty vector control strains, and a further reduction in uronic acid production in the 52K10 strain relative to WT and ∆*chaB* strains (Fig. 5b). The complemented strain (∆*chaB::*ChaB*)* was assessed by uronic acid assay; however, despite detection of the protein by Western blot and optimization of the protein production by arabinose titration, we were unable to recapitulate the WT phenotype (Supp. Figure 1). Further analysis by whole genome assembly confirmed the gene deletion of only *chaB* in the mutant strain relative to WT, suggesting precise regulation of endogenous *chaB* levels in WT, not achieved in the complemented strain (Supp. Figure 2). Notably, we observed a significant difference in capsule production between the empty vector and complement strain (*p*-value = 0.01), indicating a measurable effect of ChaB upon complementation, despite an inability to revert endogenous levels. Taken together, deletion of *chaB* reduces capsule production in *K. pneumoniae*, but does not eliminate it, suggesting a role for ChaB in regulation of capsule-associated genes.

### Characterization of *chaB* and its role in bacterial growth

**Fig. 6:**
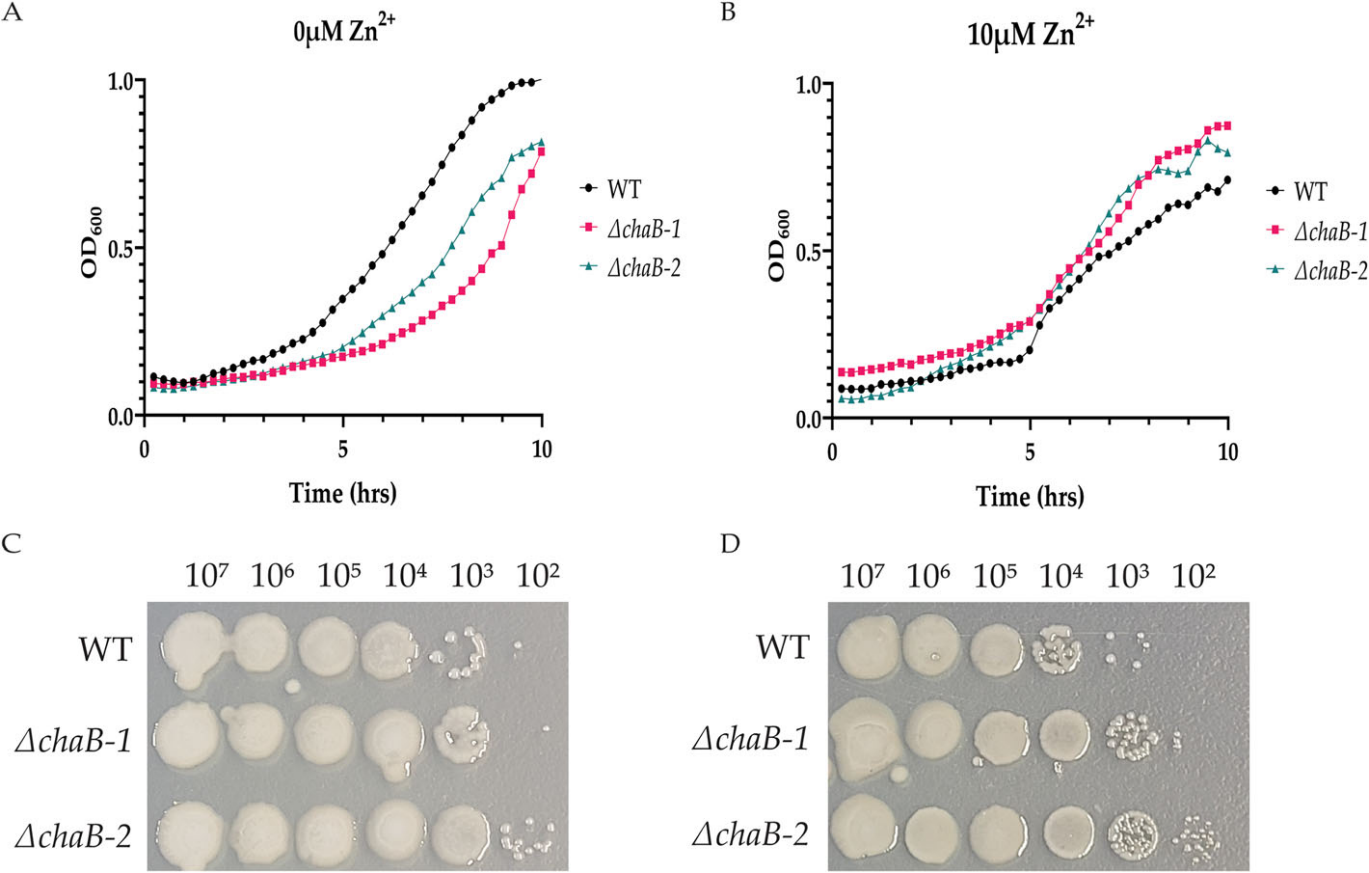
Growth analysis of *K. pneumoniae* ∆*chaB.* **a** Growth curve analysis of WT, two independent mutants of ∆*chaB* cultured in zinc-limited (0 μM). **b** Growth curve analysis of WT, two independent mutants of ∆*chaB* cultured in zinc-replete (10 μM). **c** Zinc utilization plates. WT and two independent mutants of ∆*chaB* strains grown on either limited (0 μM) plates after 72 h incubation at 37 °C. **d** Zinc utilization plates. WT and two independent mutants of ∆*chaB* strains grown on either replete (10 μM) plates after 72 h incubation at 37 °C. Experiment was performed in biological triplicate and two technical replicates

Next, we assessed differences in growth between the WT and ∆*chaB* strains under limited and replete conditions. Here, we observed the WT strain grew at a faster rate than the ∆*chaB* strains under zinc-limited (0 μM) liquid culture (Fig. 6a). We observed all strains reaching similar OD_600nm_ by stationary phase. We attribute this difference in bacterial growth to two factors: i) the mutant strains are unable to obtain the required nutrients from the minimal medium at the same rate as WT; ii) the difference in capsule production between WT and ∆*chaB* strains is detectable with the OD_600nm_ measurement. Conversely, in the presence of zinc (10 μM), the ∆*chaB* strains grow at a slightly higher rate than WT (Fig. 6b), suggesting an ability of the mutant strains to thrive in a potentially cytotoxic environment as excess zinc ions may not be transported into the cell at the same rate as WT.

We also evaluated zinc-utilization of the strains on solid agar plates (Fig. 6c & d). Here, we observed an increase in capsule production among all strains under limited vs. replete conditions, with a reduction in capsule mucoidy in the ∆*chaB* strains, corresponding with our observed microscopy and uronic acid assay results. This observation also supports the difference in growth between WT and ∆*chaB* strains under limited conditions in liquid culture to be likely influenced by capsule production. In addition, we observed that the ∆*chaB* strains produced more colonies than WT under all tested conditions, opposing our observation in the minimal medium liquid culture. These data suggest a potential difference in metal ion availability or zinc utilization between the liquid vs. solid medium. Finally, we observed a reduction in overall growth and capsule production in the replete conditions, further supporting a potential cytotoxic effect of elevated metal ion. Taken together, our data support a role for ChaB in capsule production and metal ion homeostasis in vitro. However, given the differences in observed growth in liquid culture compared to zinc utilization on solid agar, we propose future experiments to further tease apart the impact of ChaB on bacterial growth by expanding the medium conditions tested and assessing impacts on growth within a host (e.g., macrophage) environment.

### Characterization of *chaB* and its role in bacterial virulence

**Fig. 7:**
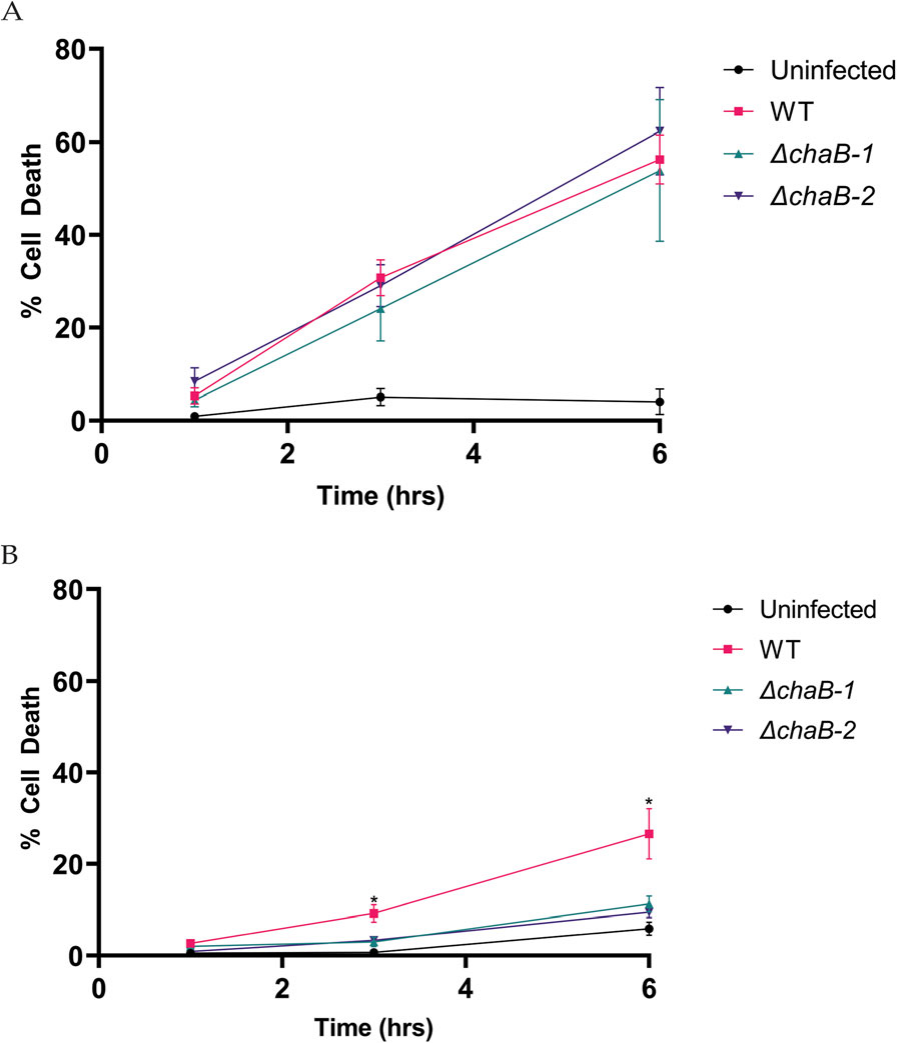
In vitro macrophage cytotoxicity assay of *K. pneumoniae.* **a** Cytotoxicity assay of Balb/C macrophages co-cultured with *K. pneumoniae* WT and ∆*chaB* following initial bacterial culturing in TSB media (nutrient rich). **b** Cytotoxicity assay of Balb/C macrophages co-cultured with *K. pneumoniae* WT and ∆*chaB* following initial bacterial culturing in M9 media (nutrient poor). Two independent deletion mutants of *chaB* were characterized across all experiments. Results reflect the average of three biological replicates and technical duplicates. Error bars depict standard deviation. *denotes significant difference between WT and mutant strains by Student’s *t*-test, *p-value* ≤ 0.05

The capsule is a known virulence factor for *K. pneumoniae* and to test the impact of *chaB* deletion on virulence, we co-cultured immortalized macrophages (Balb/C) with WT or ∆*chaB* strains. Using a cytotoxicity assay, we measured the release of lactate dehydrogenase (LDH) from macrophages across three time points. To properly assess a role for ChaB in promoting metal ion homeostasis during nutrient limitation in the host, both strains were grown in rich (TSB) or limited (M9) media prior to co-culture with macrophages. WT and ∆*chaB K. pneumoniae* strains grown under rich media prior to co-culture induced a rapid cytotoxic response in the macrophage, resulting in > 50% host cell death by 6 h of incubation (Fig. 7a). There were no measurable differences in LDH levels between macrophage co-cultured with the WT and ∆*chaB* strains over the time course of infection. In comparison, WT and ∆*chaB* strains grown under limited media prior to co-culture with macrophage resulted in reduced host cell death in the presence of the ∆*chaB* strain over 6 h of incubation (Fig. 7b). In both conditions, uninfected macrophages displayed a gradual increase in cell death, reaching approximately 5% following 6 h post mock infection, representative of natural cell death over time. Taken together, these data demonstrate a difference in macrophage cell death associated with deletion of *chaB*, implicating the gene in bacterial virulence directly connected to nutrient limited conditions.

## Discussion

Zinc availability is regulated by the host to both restrict bacterial growth (e.g., nutritional immunity) and to promote metal toxicity as defense mechanisms during pathogen infection. Therefore, maintenance of intracellular levels of zinc for pathogenic bacteria such as *K. pneumoniae* are important for growth and survival under infection conditions. In this study, we present the first in-depth proteomic profile of the impact of zinc availability on *K. pneumoniae*. Our study identified cellular and secreted proteins whose abundances were altered by the presence or absence of zinc. We analyzed the *hut* operon to validate our proteomics data and characterized a predicted cation transport regulator, ChaB, to define a novel role in zinc homeostasis, capsule regulation, and virulence under nutrient-limiting conditions. Overall, we uncover a new strategy used by the bacterium to adapt to a nutrient limited environment and propose a novel role for ChaB in regulating capsule.

We identified HutC (transcriptional repressor) with the largest increase in fold difference under replete conditions in our cellular proteome profiling. In the Gram-negative bacterium, *Acinetobacter baumannii,* histidine can chelate free zinc, and within nutrient-limited environments, the bacteria express components of the *hut* operon to promote histidine degradation, thereby releasing free zinc into the environment [36]. Given our observation of the altered abundance of HutC in the presence of zinc, our data supports a role of HutC in *K. pneumoniae* associated with zinc acquisition. This is further supported through our gene expression analysis, where we discovered that members of the *hut* operon (*hutG, hut H, hutI, hutT, and hutU*) exhibited reduced transcript expression under replete conditions associated with an increase in abundance of HutC and zinc availability. Mimicking the observation in *A. baumannii*, where the *hut* operon was active under limited conditions to possibly promote zinc release from histidine. This proposes a putative mechanism to combat host defense responses that was identified in *A. baumannii* and may also exist in *K. pneumoniae*.

A putative regulator of the Na^+^/H^+^ antiporter ChaA in *E. coli*, ChaB was characterized structurally using NMR for the identification of flexible regions capable of binding to divalent metal cations [37]. ChaA and ChaB are members of an operon, along with ChaC, proposed to be another regulator of ChaA (Oshima 1996). ChaA is inhibited by divalent cations, tying to the putative role of ChaB involvement in cation transport regulation. While many homologs exist in other bacteria such as *Shigella flexeri* and *Trabulsiella guamensis* (by BLAST search), a functional analysis has not been performed on ChaB, and moreover, its connection to metal ion availability is unknown. Here, characterization of the ∆*chaB* strain demonstrated a growth curve profile under replete conditions that mimicked the WT strain under limited conditions. Zinc can be transported by cells through an ion gradient such as calcium or sodium, to generate zinc gradients [38–40]. We propose that ChaB may play a role in the export of zinc and other cations into the cells. Whereupon, under replete conditions, WT *K. pneumoniae* displays delayed growth due to excess zinc, which may have a slight cytotoxic effect on the cells, suggesting a role for ChaB in importing ions and hence, protecting the mutant strain from excess intracellular metal concentrations. However, the ∆*chaB* strain displayed delayed growth compared to WT strain, under limited conditions, indicating an adverse response to low environmental zinc concentrations. It should be noted that deletion of *chaB* may result in unregulated ChaA activity that may have adverse effects on bacterial growth and warrants further investigation.

Our observation of reduced capsule production in ∆*chaB* relative to WT may explain why the growth curve under limited conditions showed slower growth in ∆*chaB.* The early onset and increased production of capsule in the WT could lead to the appearance of rapid growth when using OD_600nm_ values as an indicator of growth. This phenomenon has been previously reported in an acapsular mutants displaying slower growth compared to WT strains in *Streptococcus pneumoniae* [41, 42]. We hypothesize that the reduction in capsule production is not a direct correlation to the deletion of *chaB*, but instead, the impaired ability to regulate pH and osmolarity caused unregulated ChaA activity may trigger downstream effects on capsule production. We also propose additional functional analysis of the ∆*chaB* strain may offer better understanding into the characterized phenotype. Moreover, we speculate that as a putative regulator, ChaB may play a role in the regulation of downstream transcription factors involved in capsule synthesis. We suggest future experimentation into identifying the global impact of loss of *chaB* on protein production and transcriptional regulation.

To assess virulence, we co-cultured macrophages with the WT and ∆*chaB* strains. Macrophages are found ubiquitously throughout the body and are often an early responder to bacterial infection. The interaction between macrophages and *K. pneumoniae* present a well-studied system to explore the relationship between host and pathogen under in vitro settings [43–46]. Macrophage cytotoxicity assays showed that deletion of *chaB* (leading to reduced capsule size) impacts bacterial virulence. The reduced virulence was only apparent upon initial bacterial culturing in minimal media; conditions that were found to induce increased capsule production in comparison to rich medium. Previous findings have reported that upon phagocytosis, *K. pneumoniae* survives in a vacuolar compartment where it triggers programmed cell death of macrophages [43]. Furthermore, once internalized, *K. pneumoniae* reduced the expression of capsule biosynthesis genes. While survival of macrophage is unaffected in rich medium between the strains, during bacterial growth in minimal medium (to induce capsule production), the ∆*chaB* strain may be more susceptible to phagocytosis, due to its reduced size as the capsule is an important virulence factor protecting the bacteria from phagocytosis [47]. Moreover, the difference in size between WT cells producing a capsule and ∆*chaB*, which shows a reduced capsule, may influence the number of phagocytosed cells, suggesting that virulence of *K. pneumoniae* is influenced by both capsule production and number of internalized cells. In addition, we propose that the role of ChaB in promoting metal ion homeostasis (as observed in the growth curves and plate assays) is evident by a reduction in virulence over the time course of infection, suggesting that the bacterium lacking *chaB* is unable to adapt to the nutrient-limited environment. The decreased virulence reported in ∆*chaB* prompts the need for follow-up studies to further characterize the relationship between ChaB and the host, as well as ascertaining the potential of ChaB as a target for antimicrobial compounds.

## Conclusions

In summary, we used quantitative proteomics to investigate the first analysis of zinc availability on the proteome and secretome of *K. pneumoniae*. We identified proteins that display a significant change in abundance in the presence or absence of zinc, including a transcriptional repressor and putative cation transport regulator. We validated our proteomic findings by performing gene expression analysis on the *hut* operon, confirming repression of its target genes under replete conditions. In addition, we identified a cation transport regulator (ChaB) under replete conditions and characterized its role in capsule production, growth, and virulence of *K. pneumoniae*. Moreover, we identified a novel morphological difference – a reduction in capsule size – associated with the absence of the *chaB* that may provide insight into a novel role in zinc homeostasis that impact its cytotoxic effects during an infection setting. Further studies are required to identify either a direct or indirect relationship between ChaB and capsule production.

## Methods

### Bacterial cells and growth conditions

*K. pneumoniae* K52145, a hypervirulent WT strain and 52K10, an acapsular mutant strain (generously provided by Dr. Arturo Zychlinsky, Max Planck Institute for Infection Biology), was used in the experiments. Bacterial strains were cultured in Tryptic Soy broth (TSB) (Sigma-Aldrich) or grown on Tryptic Soy agar (TSA). Zinc-limited medium was produced by first passing milliQ water through Chelex-100 resin (Bio-Rad). Chelex-treated milliQ water was used to prepare M9 minimal media. M9 minimal salts (5X) (Fisher Scientific) were prepared according to manufacturer’s instructions, followed by supplementation with 20% glucose, 1 M MgSO_4_ and 1 M CaCl_2_. Zinc-replete medium was produced by supplementing the limited media (0 μM) with ZnSO_4_ to a final concentration of 10 μM. *K. pneumoniae* was grown at 37 °C with 200 rpm shaking.

### Sample preparation for mass spectrometry analysis

WT *K. pneumoniae* was cultured in TSB overnight, cells were collected, washed with limited-media, and sub-cultured into either zinc-limited or -replete media. Each condition included four biological replicates. Samples were grown until cells reached mid-log phase, upon which, total proteome analysis was performed as previously described [9]. Briefly, cells were collected, washed twice with phosphate-buffered saline (PBS), and resuspended in cold 100 mM Tris-HCl (pH 8.5). Cells were lysed using a probe sonicator set to five cycles of 30 sec on/off at an amplitude of 30%. Samples were treated with 2% sodium dodecyl sulphate and 10 mM dithiothreitol (DTT). Samples were heated to 95 °C prior to treatment with 55 mM iodoacetamide (IAA). Proteins were submitted to acetone precipitation, followed by digestion of proteins using LysC and trypsin overnight at room temperature. Digested peptides were purified and desalted using STop And Go Extraction (STAGE) tips [48].

For secretome analysis, the culture supernatant was filtered through a 0.22 μM syringe filter and then performed as previously described [9]. For each sample, 500 μl of filtered supernatant was treated with DTT, IAA, followed by digestion using LysC and trypsin. Digested peptides were desalted and purified as described above.

### Mass spectrometry

Digested peptides were resuspended in 12 μl buffer A (0.1% TFA). Six μl of each sample was analyzed on a Q Exactive™ HF-X hybrid quadrupole-orbitrap mass spectrometer (ThermoFisher Scientific) coupled to an Easy-nLC™ 1200 High-Performance Liquid Chromatography (ThermoFisher Scientific). Samples were loaded onto an in-line 75 μm × 50 cm PepMap RSLC EASY-Spray column filled with 2 μm C18 reverse-phase silica beads (ThermoFisher Scientific). Peptides were separated and directly electrosprayed into the mass spectrometer using a linear gradient from 3 to 20% Buffer B over 18 min, from 20 to 35% Buffer B over 31 mins, followed by a steep 2 min ramp to 100% Buffer B for 9 min in 0.1% Formic acid at a constant flow of 250 nl/min. The mass spectrometer was operated in data-dependent mode, switching automatically between one fill scan and subsequent MS/MS scans of 30 most abundant peaks, with full-scans (*m/z* 400–1600) acquired in the Orbitrap analyzer with a resolution of 60,000 at 400 m/z.

### Data analysis and bioinformatic processing

Raw mass spectrometry files were analyzed using MaxQuant (ver. 1.6.0.26) [49]. The spectra were searched using the Andromeda search engine against the UniProt *K. pneumoniae* proteome (*Klebsiella pneumoniae subsp. pneumoniae* (strain ATCC 700721 / MGH 78578), accessed Dec. 2018 with 5127 sequences) [50]. For the search, cysteine carbomethylation was set as a fixed modification, while N-acetylation of proteins and oxidation of methionine were set as variable modifications. Additional parameters include: trypsin digestion, a minimum peptide length of seven amino acids, and allowing up to two missed cleavages. For protein identification, we required a minimum of two peptides and set the FDR to 1%. The ‘match between runs’ feature of MaxQuant was used. Quantification was performed by label-free quantification (LFQ) using the MaxLFQ algorithm [51]. The raw data is deposited in the PRIDE partner repository for the ProteomeXchange Consortium (Data set identifier = PXD016042). All mass spectrometry experiments were performed in biological quadruplicate and technical duplicate.

Downstream statistical analysis and data processing was performed using Perseus (ver. 1.6.2.2) [52]. Further statistical analysis was only performed on proteins that were present in at least three of four replicates within each sample condition. Upon filtering, missing values were imputed based on a normal distribution. A Student’s *t*-test was performed to identify significantly different proteins (*p*-value ≤0.05). The Benjamini-Hochberg multiple hypothesis correction testing (FDR = 0.05) was applied [53]. For 1D annotation enrichment, a two-sample test was performed using a Student’s *t*-test with *p*-value ≤0.05, FDR = 0.05 and score < − 0.5, < 0.5. Data visualization was produced using RStudio.

### Gene expression analysis

Total RNA was extracted from WT *K. pneumoniae* cultured in zinc-limited or -replete media using the PureLink™ RNA Mini Kit (Invitrogen). Extracted RNA was treated with DNaseI (ThermoFisher Scientific). DNase-treated RNA was reverse-transcribed using High Capacity cDNA Reverse Transcription Kit (Applied BioSystem). For qRT-PCR analysis, 2X PerfeCta SYBR Green FastMix, ROX (Quanta BioScience) was combined with gene-specific primers (Supp. Table 1). Reactions were analyzed using StepOnePlus Real Time PCR system (ThermoFisher Scientific). All reactions were performed in three biological replicates and two technical replicates, and expression was normalized to the reference genes, *recA* and *rho* [54].

### Construction of ∆*chaB* deletion mutant

Mutation of *K. pneumoniae* was produced following the lambda red recombinase protocol [55]. To summarize, electrocompetent WT *K. pneumoniae* was transformed with the pSim6 plasmid, which contains the lambda red machinery [56]. Primers were designed (Supp. Table 1) homologous to the regions upstream and downstream of *chaB* to amplify the chloramphenicol resistance marker from the pKD3 plasmid. Subsequently, *K. pneumoniae*-pSim6 was transformed with the PCR product and plated on TSA containing 15 μg/ml of chloramphenicol. Deletion strains were confirmed by PCR and sequencing, followed by incubation at 37 °C to cure the pSim6 plasmid.

### Complementation of ∆*chaB*

Gene deletion mutant was complemented by construction of vector containing the full-length copy of *chaB* under the control of an arabinose-inducible promoter as previously described [9]. The gene was cloned to produce a FLAG-tagged ChaB fusion protein upon expression. The full-length gene was amplified from *K. pneumoniae* gDNA with restriction site overhangs of NcoI and XbaI. The gene product was cloned into the pBAD24 vector using restriction-based cloning and transformed into *E. coli* TOP10 cells [57]. Following plasmid propagation and isolation, the purified plasmid was transformed into the ∆*chaB* deletion strain.

### Protein complementation and Western blot

*K. pneumoniae* Δ*chaB*::ChaB and empty vector control (negative control) were cultured in TSB supplemented with 100 μg/mL ampicillin for selection. Cultures were supplemented with various concentrations of arabinose (e.g., 0.2, 0.1, 0.01 and 0.001%) and incubated at 37 °C for 3, 6, 9, and 12 h. At listed time points, cells were harvested. Whole cell extracts were separated by SDS-PAGE and transferred to a polyvinylidene difluoride membrane using a transfer apparatus according to the manufacturer’s protocols (Bio-Rad). After incubation with 3% non-fat milk in 1X TBS (50 mM Tris, 150 mM NaCl, pH 7.5) overnight at 4 °C, the membrane was washed five times with TBST (1X TBS, 0.05% Tween-20), and incubated with Monoclonal ANTI-FLAG® M2 antibody (Sigma-Aldrich) for 1 h. Membranes were washed three times for 5 min and incubated with 1:3000 dilution of horseradish peroxidase-conjugated Goat anti-mouse IgG Fc secondary antibody (Invitrogen) for 1 h. Blots were washed with TBST three times and developed using the Clarity Max Western ECL Substrates system (BioRad). Experiment was performed in biological and technical duplicates.

### Genomic DNA extraction, Illumina sequencing and whole genome analysis

Genomic DNA was extracted from *K. pneumoniae* WT and two independent ∆*chaB* mutants using PureLink™ Genomic DNA Mini Kit (ThermoFisher Scientific). Purified gDNA were prepared using an Illumina Nextera kit by the Microbial Genome Sequencing Center (Pennsylvania, USA) followed by Illumina sequencing on a NextSeq 550 platform. Raw .fastq files were processed using Geneious Prime 2021.0.2 (geneious.com) [58]. *K. pneumoniae* WT files were initially trimmed using an in-suite BBDuk plug-in to trim adaptors and low-quality reads [59]. The trimmed reads were mapped to a reference genome retrieved from NCBI. Correct assembly was validated by searching for variations (i.e., deletion or insertion) and SNPs. Upon confirmation of correct assembly, the independent deletion mutants were mapped to the assembled WT genome. To identify any non-specific insertions of chloramphenicol into the *K. pneumoniae* mapping settings were modified to search for insertions or deletions up to 1100 bp (matching the expected size of the chloramphenicol gene). Whole genome alignment between the WT and ∆*chaB* strain was performed using a progressive Mauve alignment from the Mauve plug-in using default alignment settings [60].

### Capsule morphology

To visualize changes in capsule production between the WT and ∆*chaB* strains of *K. pneumoniae*, cultures were set as above, and at mid-log phase of growth, culture aliquots were collected and stained with India Ink (Hardy Diagnostic). Cells were visualized on a Zeiss Axiovert 200 M microscope using DIC and images were captured using a Hamamatsu ORCA-R2 digital camera. Volocity (ver. 6.3) software (Quorum Technology) was used for image capture and processing.

### Uronic acid assay for capsule quantification

The amount of *Klebsiella* capsule present was quantified following previously described protocols [34, 35, 61, 62]. Briefly, 500 μL of overnight culture (M9 minimal media, 37 °C with shaking) was incubated with 100 μL of capsule extraction solution (500 mM citric acid pH 2.0, with 1% Zwittergent 3–10 (Sigma-Aldrich)) for 20 mins at 50 °C with shaking. Following centrifugation at 16000×g for 5 mins to pellet cellular debris. Supernatant (300 μL) was precipitated in 1.5 mL of pure ethanol on ice for 30 mins. The capsule extract was pelleted by centrifugation at 16000×g for 20 mins at 4 °C, dried at 37 °C with shaking for 10 min, and then resuspended in 200 μL sterile water. Tetraborate solution (12.5 mM disodium tetraborate in concentrated sulfuric acid) was added to each sample on ice, cooled for 10 mins, vortexed vigorously, heated at 99 °C for 5 mins, and then chilled on ice immediately for a minimum of 5 mins. Twenty μL of phenylphenol solution (0.15% 2-Hydroxybiphenyl (Sigma-Aldrich) in 0.5% NaOH) was added to each sample and vigorously vortexed before measuring absorbance at OD_520nm_. Uronic acid concentration was calculated using a glucuronic acid (Sigma) standard curve and OD_600nm_ measurements were taken for each culture prior to capsule extraction and normalized, as needed.

### Growth curve analysis

To determine the impact of deleting *chaB* on growth, WT and mutant strains of *K. pneumoniae* were cultured overnight in TSB prior to sub-culturing into zinc-limited or -replete media. Cultures were grown at 37 °C with 200 rpm shaking. Growth was monitored through OD_600nm_ measurements taken every 15 min in a Synergy H1 microplate reader over 10 h (BioTek). Two individual mutants of ∆*chaB* were monitored in five biological replicates and two technical replicates. Growth curves were confirmed by growth in shaker flasks at 37 °C with 200 rpm shaking.

### Zinc utilization plates

To examine the impact of zinc on colony morphology and growth, WT and ∆*chaB* strains of *K. pneumoniae* were initially grown in TSB overnight. Cells were collected, washed, and resuspended in zinc-limited media. Cells were plated on Chelex-treated M9 minimal media plates supplemented with 10 μM ZnSO_4_ or plates without zinc. Cells were plated as a serial dilution starting from 1 × 10^7^ to 1 × 10^2^ colony forming units (CFU). Each dilution series was set with two biological and two technical replicates and performed twice. Two individual mutants of ∆*chaB* were plated. Plates were incubated at 37 °C.

### Macrophage cell culture

Immortalized murine macrophages originally derived from WT Balb/C mice (generously provided by Dr. Felix Meissner, Max Planck Institute of Biochemistry) were grown in Dulbecco’s Modified Eagle Medium (DMEM) supplemented with 10% heat-inactivated fetal bovine serum (FBS), 2 mM L-glutamine and 1% Pen/Strep. Cells were grown at 37 °C in a humidified 5% CO_2_ atmosphere.

### Infection of macrophages

To test the impact of deleting *chaB* on virulence, infection of Balb/C macrophages was performed following an adapted protocol [43]. Macrophages were seeded into 12-well plates at 0.1 × 10^6^ in DMEM complete medium and grown as described above. At confluence (0.5 × 10^6^), media was exchanged with DMEM complete media without Pen/Strep. *K. pneumoniae* strains were cultured in TSB or M9, harvested, washed with PBS, and 5.0 × 10^7^ cells were added to the macrophage cells to achieve multiplicity of infection (MOI) of 100:1. After a 90-min incubation, media was removed, cells were washed with PBS, and incubated in Pen/Strep-free DMEM supplemented with gentamicin (300 μg/ml). After a 90-min gentamicin treatment, media was removed, cells were washed with PBS, and incubated in Pen/Strep-free DMEM supplemented with gentamicin (100 μg/ml). Mock infections (no *K. pneumoniae*) were set following the above protocol. Both infections and mock-infections were set in biological triplicate and technical duplicate. All incubation steps were carried out at 37 °C in a humidified 5% CO_2_ atmosphere.

### Cytotoxicity assay

At selected time points (1, 3, and 6 hpi), supernatant from *K. pneumoniae*-infected macrophages was collected. Lactate dehydrogenase (LDH) release from infected and uninfected macrophages was colourimetrically measured (OD_490nm_) using the CytoTox 96® Non-Radioactive Cytotoxicity Assay (Promega) according to manufacturer’s instructions. Cytotoxicity was calculated according to manufacturer’s instructions. Supernatant cell death values were normalized to total cell lysate OD_490nm_ measurements.

## Supplementary Information

**Additional file 1.**

**Additional file 2.**

**Additional file 3.**

**Additional file 4.**

## Data Availability

The mass spectrometry proteomics data have been deposited in the PRIDE partner repository for the ProteomeXchange Consortium with the data set identifier: PXD016042.

## Abbreviations

WT: Wild type
PCA: Principal component analysis
FDR: False discovery rate
DIC: Differential interference contrast
LDH: Lactate dehydrogenase
TSB: Tryptic soy broth
TSA: Tryptic soy agar
PBS: Phosphate-buffered saline
DTT: Dithiothreitol
IAA: Iodoacetamide
STAGE: STop And Go Extraction
nLC: Nano liquid chromatography
MS/MS: Mass spectrometry/mass spectrometry
LFQ: Label-free quantification
DMEM: Dulbecco’s Modified Eagle Medium
FBS: Fetal bovine serum
MOI: Multiplicity of infection

## References

[1] Magill SS (2014). Multistate point-prevalence survey of health care–associated infections. N Engl J Med.

[2] Paczosa MK, Mecsas J (2016). *Klebsiella pneumoniae*: going on the offense with a strong defense. Microbiol Mol Biol Rev.

[3] Roe CC, Vazquez AJ, Esposito EP, Zarrilli R, Sahl JW (2019). Diversity, virulence, and antimicrobial resistance in isolates from the newly emerging *Klebsiella pneumoniae* ST101 lineage. Front Microbiol..

[4] Sakkas H, Bozidis P, Ilia A, Mpekoulis G, Papadopoulou C (2019). Antimicrobial resistance in bacterial pathogens and detection of carbapenemases in *Klebsiella pneumoniae* isolates from hospital wastewater. Antibiotics..

[5] Adamo R, Margarit I (2018). Fighting antibiotic-resistant *Klebsiella pneumoniae* with “sweet” immune targets. mBio.

[6] Rajapaksha RMCP, Tobor-Kapłon MA, Bååth E (2004). Metal toxicity affects fungal and bacterial activities in soil differently. Appl Environ Microbiol.

[7] Lin CT (2011). Fur regulation of the capsular polysaccharide biosynthesis and iron-acquisition systems in *Klebsiella pneumoniae* CG43. Microbiology.

[8] Chapman R, Dean ACR (1982). Action of iron and iron-complexes on *Klebsiella pneumoniae (Klebsiella aerogenes)*. Folia Microbiol (Praha).

[9] Muselius B, Sukumaran A, Yeung J, Geddes-McAlister J (2020). Iron limitation in *Klebsiella pneumoniae* defines new roles for Lon protease in homeostasis and degradation by quantitative proteomics. Front Microbiol..

[10] Bachman MA, Lenio S, Schmidt L, Oyler JE, Weiser JN (2012). Interaction of lipocalin 2, transferrin, and siderophores determines the replicative niche of *Klebsiella pneumoniae* during pneumonia. MBio.

[11] Holden VI, Breen P, Houle S, Dozois CM, Bachman MA (2016). *Klebsiella pneumoniae* Siderophores induce inflammation, bacterial dissemination, and HIF-1α stabilization during pneumonia. MBio.

[12] Holden VI (2018). Iron Acquisition and Siderophore Release by Carbapenem-Resistant Sequence Type 258 *Klebsiella pneumoniae*. mSphere.

[13] Andreini C, Banci L, Bertini I, Rosato A (2006). Zinc through the three domains of life. J Proteome Res.

[14] McCall KA, Huang C, Fierke CA (2000). Function and mechanism of zinc metalloenzymes. J Nutr.

[15] Hood MI, Skaar EP (2012). Nutritional immunity: transition metals at the pathogen-host interface. Nat Rev Microbiol.

[16] Mikhaylina A, Ksibe AZ, Scanlan DJ, Blindauer CA (2018). Bacterial zinc uptake regulator proteins and their regulons. Biochem Soc Trans.

[17] Poole RK (2009). Severe zinc depletion of *Escherichia coli*. J Biol Chem.

[18] Choi SH (2017). Zinc-dependent regulation of zinc import and export genes by zur. Nat Commun.

[19] Panina EM, Mironov AA, Gelfand MS (2003). Comparative genomics of bacterial zinc regulons: enhanced ion transport, pathogenesis, and rearrangement of ribosomal proteins. Proc Natl Acad Sci.

[20] Dow A, Prisic S (2018). Alternative ribosomal proteins are required for growth and morphogenesis of *Mycobacterium smegmatis* under zinc limiting conditions. PLoS One.

[21] Eide DJ (2006). Zinc transporters and the cellular trafficking of zinc. Biochim Biophys Acta, Mol Cell Res.

[22] Wilson D, Citiulo F, Hube B. Zinc exploitation by pathogenic fungi. PLoS Pathog. 2012. 10.1371/journal.ppat.1003034. 10.1371/journal.ppat.1003034 PMC3534374 23308062

[23] Wang D, Fierke C (2013). A. the BaeSR regulon is involved in defense against zinc toxicity in E. coli. Metallomics.

[24] Stocks CJ (2019). Uropathogenic *Escherichia coli* employs both evasion and resistance to subvert innate immune-mediated zinc toxicity for dissemination. Proc Natl Acad Sci U S A.

[25] Neyrolles O, Mintz E, Catty P (2013). Zinc and copper toxicity in host defense against pathogens: *Mycobacterium tuberculosis* as a model example of an emerging paradigm. Front Cell Infect Microbiol.

[26] Ong CLY, Berking O, Walker MJ, McEwan AG (2018). New insights into the role of zinc acquisition and zinc tolerance in group a streptococcal infection. Infect Immun.

[27] Sukumaran A, Woroszchuk E, Ross T, Geddes-McAlister J. Proteomics of host-bacterial interactions: new insights from dual perspectives. Can J Microbiol. 2020:1–43. 10.1139/cjm-2020-0324. 10.1139/cjm-2020-0324 33027598

[28] Smith SM, Wunder MB, Norris DA, Shellman YG (2011). A simple protocol for using a LDH-based cytotoxicity assay to assess the effects of death and growth inhibition at the same time. PLoS One..

[29] Desrosiers DC (2010). Znu is the predominant zinc importer in *Yersinia pestis* during in vitro growth but is not essential for virulence. Infect Immun.

[30] Velasco E (2018). A new role for zinc limitation in bacterial pathogenicity: modulation of α-hemolysin from uropathogenic *Escherichia coli*. Sci Rep.

[31] Cox J, Mann M. 1D and 2D annotation enrichment: a statistical method integrating quantitative proteomics with complementary high-throughput data. BMC Bioinformatics. 2012. 10.1186/1471-2105-13-S16-S12. 10.1186/1471-2105-13-S16-S12 PMC3489530 23176165

[32] Bender RA (2012). Regulation of the histidine utilization (hut) system in bacteria. Microbiol Mol Biol Rev.

[33] Lee JC, et al. *Klebsiella pneumoniae* secretes outer membrane vesicles that induce the innate immune response. FEMS Microbiol Lett. 2012. 10.1111/j.1574-6968.2012.02549.x. 10.1111/j.1574-6968.2012.02549.x 22428779

[34] Blumenkrantz N, Asboe-Hansen G (1973). New method for quantitative determination of uronic acids. Anal Biochem.

[35] Walker KA (2019). A *Klebsiella pneumoniae* regulatory mutant has reduced capsule expression but retains hypermucoviscosity. MBio.

[36] Capdevila DA, Wang J, Giedroc DP (2016). Bacterial strategies to maintain zinc metallostasis at the host-pathogen interface. J Biol Chem.

[37] Osborne MJ, Siddiqui N, Iannuzzi P, Gehring K (2004). The solution structure of ChaB, a putative membrane ion antiporter regulator from *Escherichia coli*. BMC Struct Biol.

[38] Sekler I, Sensi SL, Hershfinkel M, Silverman WF (2015). Mechanism and regulation of cellular zinc transport. Mol Med.

[39] Sensi SL (1997). Measurement of intracellular free zinc in living cortical neurons: routes of entry. J Neurosci.

[40] Ohana E (2004). A sodium zinc exchange mechanism is mediating extrusion of zinc in mammalian cells. J Biol Chem.

[41] Hathaway LJ (2012). Capsule type of *Streptococcus pneumoniae* determines growth phenotype. PLoS Pathog..

[42] Bättig P, Mühlemann K (2007). Capsule genes of *Streptococcus pneumoniae* influence growth in vitro. FEMS Immunol Med Microbiol.

[43] Cano V, et al. *Klebsiella pneumoniae* survives within macrophages by avoiding delivery to lysosomes. Cell Microbiol. 2015. 10.1111/cmi.12466. 10.1111/cmi.12466 26045209

[44] Guilhen C (2019). Colonization and immune modulation properties of *Klebsiella pneumoniae* biofilm-dispersed cells. NPJ Biofilms Microbiomes..

[45] Ares MA (2019). The interaction of *Klebsiella pneumoniae* with lipid rafts-associated cholesterol increases macrophage-mediated phagocytosis due to down regulation of the capsule polysaccharide. Front Cell Infect Microbiol..

[46] Sukumaran A, et al. Decoding communication patterns of the innate immune system by quantitative proteomics. J Leukoc Biol. 2019. 10.1002/JLB.2RI0919-302R. 10.1002/JLB.2RI0919-302R PMC7309189 31556465

[47] Domenico P, Salo RJ, Cross AS, Cunha BA (1994). Polysaccharide capsule-mediated resistance to opsonophagocytosis in *Klebsiella pneumoniae*. Infect Immun.

[48] Rappsilber J, Mann M, Ishihama Y (2007). Protocol for micro-purification, enrichment, pre-fractionation and storage of peptides for proteomics using StageTips. Nat Protoc.

[49] Cox J, Mann M (2008). MaxQuant enables high peptide identification rates, individualized p.p.b.-range mass accuracies and proteome-wide protein quantification. Nat Biotechnol.

[50] Cox J, et al. Andromeda: a peptide search engine integrated into the MaxQuant environment. J Proteome Res. 2011. 10.1021/pr101065j. 10.1021/pr101065j 21254760

[51] Cox J (2014). Accurate proteome-wide label-free quantification by delayed normalization and maximal peptide ratio extraction, termed MaxLFQ. Mol Cell Proteomics.

[52] Tyanova S (2016). The Perseus computational platform for comprehensive analysis of (prote)omics data. Nat Methods.

[53] Benjamini Y, Hochberg Y. Controlling the false discovery rate: a practical and powerful approach to multiple testing. J R Stat Soc Ser B. 1995. 10.1111/j.2517-6161.1995.tb02031.x.

[54] Gomes AÉI (2018). Selection and validation of reference genes for gene expression studies in *Klebsiella pneumoniae* using reverse transcription quantitative real-time PCR. Sci Rep.

[55] Datsenko KA, Wanner BL. One-step inactivation of chromosomal genes in *Escherichia coli* K-12 using PCR products. Proc Natl Acad Sci. 2000. 10.1073/pnas.120163297. 10.1073/pnas.120163297 PMC18686 10829079

[56] Datta S, Costantino N, Court DL (2006). A set of recombineering plasmids for gram-negative bacteria. Gene.

[57] Guzman LM, Belin D, Carson MJ, Beckwith J (1995). Tight regulation, modulation, and high-level expression by vectors containing the arabinose PBAD promoter. J Bacteriol.

[58] Kearse M (2012). Geneious basic: an integrated and extendable desktop software platform for the organization and analysis of sequence data. Bioinformatics.

[59] Bushnell B, Rood J, Singer E (2017). BBMerge – accurate paired shotgun read merging via overlap. PLoS One.

[60] Darling ACE, Mau B, Blattner FR, Perna NT. Mauve: multiple alignment of conserved genomic sequence with rearrangements. Genome Res. 2004. 10.1101/gr.2289704. 10.1101/gr.2289704 PMC442156 15231754

[61] Domenico P, Schwartz S, Cunha BA. Reduction of capsule polysaccharide production in *Klebsiella pneumoniae* by sodium salicylate. Infect Immun. 1989;57(12): 3778–82. https://pubmed.ncbi.nlm.nih.gov/2680983/. 10.1128/iai.57.12.3778-3782.1989 PMC259904 2680983

[62] Dorman MJ, Feltwell T, Goulding DA, Parkhill J, Short FL. The capsule regulatory network of *Klebsiella pneumoniae* defined by density-TraIDSort. MBio. 2018;9(6): e01863–18. https://www.ncbi.nlm.nih.gov/pmc/articles/PMC6247091/#textS1. 10.1128/mBio.01863-18 PMC6247091 30459193

