# Supplementary material for: Zinc limitation in Klebsiella pneumoniae profiled by quantitative proteomics influences transcriptional regulation and cation transporter-associated capsule production
Source: BMC Microbiol. 2021 Feb 10;21:43. doi: 10.1186/s12866-021-02091-8 (PMC7874612; doi:10.1186/s12866-021-02091-8)
Supplement: Supplementary file 1 — Additional file 1. [file 12866_2021_2091_MOESM1_ESM.docx]

**Supplemental Table 1: Primer sequences for qRT-PCR analysis**

| **Gene name** | **Primer name** | **Sequence** | **Annealing Temperature (°C)** |
| --- | --- | --- | --- |
| *hutC* | hutC-F | TCACTCGCTGATGGTGCATT | 60 |
|  | hutC-R | ACAGGTAATCATGGGGCGTG | 60 |
| *hutG* | hutG-F | GCATCTGGATCTGCGCTTTG | 60 |
|  | hutG-R | GCTAACCCCGATGCAGGTAT | 60 |
| *hutH* | hutH-F | ATATTACCCTCGACAGCGGC | 60 |
|  | hutH-R | TCTTCCGTCGAGATACGGGT | 60 |
| *hutI* | hutI-F | GCATTCCGGTAAAAGGCCAC | 60 |
|  | hutI-R | CGCCCTCTTCCGTCAGATAC | 60 |
| *hutT* | hutT-F | TGCCCGACACATTCGGTTTA | 60 |
|  | hutT-R | GCATGACGATAAACACCGCC | 60 |
| *hutU* | hutU-F | GCGCAACTGGGAATGCTATG | 60 |
|  | hutU-R | AGGTTGGAGTTGGCGATCAG | 60 |
| *recA* | recA-F | TCGGTCAGGGTAAAGCGAAC | 60 |
|  | recA-R | CGGTTTCTTCTGCGTCGTTG | 60 |
| *rho* | rho-F | TTCGTCCTCCGAAAGAGGGT | 60 |
|  | rho-R | AGTACGCGAGCGGTTAAGTC | 60 |
