# Supplementary material for: Zinc limitation in Klebsiella pneumoniae profiled by quantitative proteomics influences transcriptional regulation and cation transporter-associated capsule production
Source: BMC Microbiol. 2021 Feb 10;21:43. doi: 10.1186/s12866-021-02091-8 (PMC7874612; doi:10.1186/s12866-021-02091-8)
Supplement: Supplementary file 2 — Additional file 2. [file 12866_2021_2091_MOESM2_ESM.docx]

**Supplemental table 2: Proteins identified in the supernatant of *K. pneumoniae* under zinc-limited and -replete conditions overlapping with previous profiling of extracellular vesicles.**

| **Protein ID** | **Protein Name** |
| --- | --- |
| A6T4F8 | 30S ribosomal protein S20 |
| A6T4Q8 | Dihydrolipoamide acetyltransferase |
| A6T4X1 | 30S ribosomal protein S2 |
| A6T4Y0 | Periplasmic molecular chaperone for outer membrane proteins |
| A6T6Q8 | Outer membrane protein X |
| A6T751 | Outer membrane protein 3a |
| A6T7G4 | Putative fibronectin-binding protein |
| A6TAE1 | Murein lipoprotein |
| A6TCG5 | Serine hydroxymethyltransferase |
| A6TCL4 | 50S ribosomal protein L19 |
| A6TCV7 | Putative uncharacterized protein |
| A6TCW1 | Protein RecA |
| A6TD53 | Enolase |
| A6TED7 | Putative uncharacterized protein YqjD |
| A6TEN8 | 30S ribosomal protein S9 |
| A6TEU7 | 50S ribosomal protein L17 |
| A6TEU9 | 30S ribosomal protein S4 |
| A6TEV3 | 50S ribosomal protein L15 |
| A6TEV5 | 30S ribosomal protein S5 |
| A6TEV7 | 50S ribosomal protein L6 |
| A6TEV8 | 30S ribosomal protein S8 |
| A6TEW0 | 50S ribosomal protein L5 |
| A6TEW2 | 50S ribosomal protein L14 |
| A6TEW5 | 50S ribosomal protein L16 |
| A6TEW6 | 30S ribosomal protein S3 |
| A6TEW7 | 50S ribosomal protein L22 |
| A6TEX1 | 50S ribosomal protein L4 |
| A6TEX2 | 50S ribosomal protein L3 |
| A6TEX3 | 30S ribosomal protein S10 |
| A6TEX7 | Elongation factor Tu |
| A6TEX8 | Elongation factor EF-2 |
| A6TEX9 | 30S ribosomal protein S7 |
| A6TEY5 | Peptidyl-prolyl cis-trans isomerase |
| A6TGN5 | 50S ribosomal protein L11 |
| A6TGN6 | 50S ribosomal protein L1 |
| A6TGN8 | 50S ribosomal protein L10 |
| A6TGT4 | Glucose-6-phosphate isomerase |
| A6TH53 | 60 kDa chaperonin |
| A6THB1 | 30S ribosomal protein S6 |
| A6THB3 | 30S ribosomal protein S18 |
| A6THB4 | 50S ribosomal protein L9 |
