# Supplementary material for: Zinc limitation in Klebsiella pneumoniae profiled by quantitative proteomics influences transcriptional regulation and cation transporter-associated capsule production
Source: BMC Microbiol. 2021 Feb 10;21:43. doi: 10.1186/s12866-021-02091-8 (PMC7874612; doi:10.1186/s12866-021-02091-8)
Supplement: Supplementary file 3 — Additional file 3. [file 12866_2021_2091_MOESM3_ESM.docx]

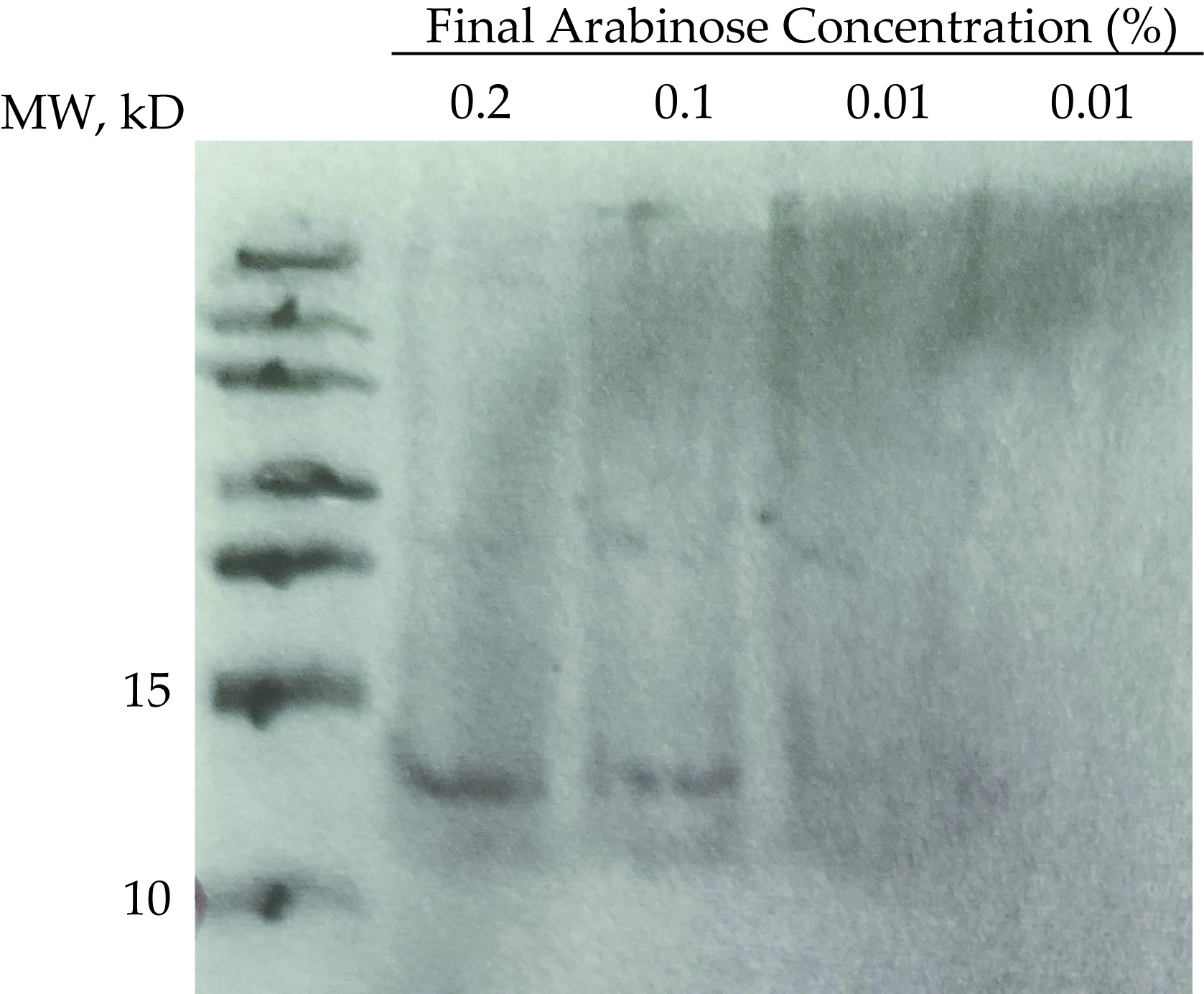


**Supplemental Figure 1. Western blot of to confirm production of ChaB.** *K. pneumoniae* △*chaB*::ChaB colonies were cultured in TSB with 0.2, 0.1, 0.01 or 0.001%, Arabinose and whole cell extract was separated using SDS-PAGE prior to blotting on a PVDF membrane. Membrane was probed using Monoclonal ANTI-FLAG® M2 antibody (Sigma-Aldrich) followed by horseradish peroxidase-conjugated Goat anti-mouse IgG Fc secondary antibody. Expected size of ChaB-FLAG fusion protein is 10.8 kD.
