# Supplementary material for: Zinc limitation in Klebsiella pneumoniae profiled by quantitative proteomics influences transcriptional regulation and cation transporter-associated capsule production
Source: BMC Microbiol. 2021 Feb 10;21:43. doi: 10.1186/s12866-021-02091-8 (PMC7874612; doi:10.1186/s12866-021-02091-8)
Supplement: Supplementary file 4 — Additional file 4. [file 12866_2021_2091_MOESM4_ESM.docx]

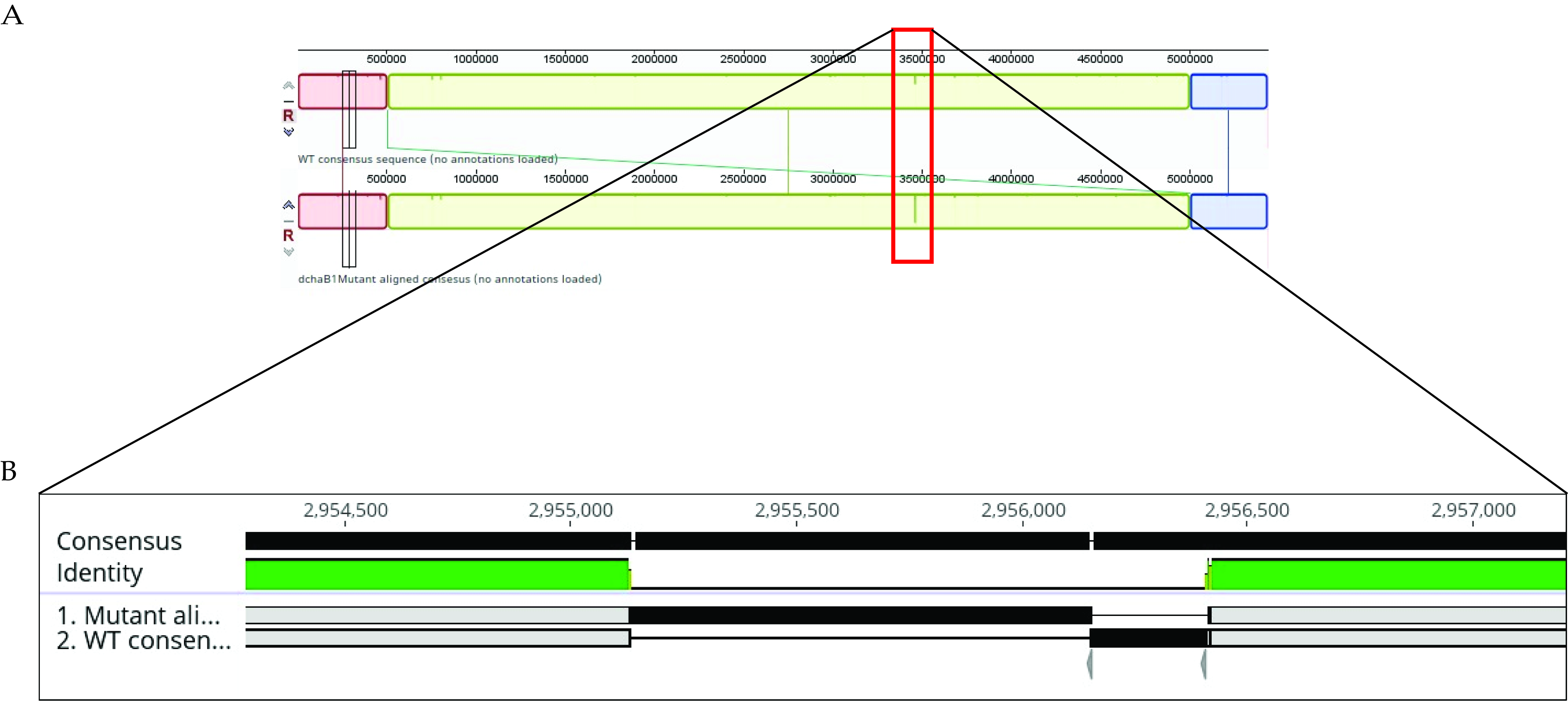


**Supplemental Figure 2. Whole genome alignment of *K. pneumoniae* WT and △*chaB* strain.** The Geneious platform was utilized to align reads and visualizing alignments following Illumina sequencing A) Whole genome alignment using Mauve viewer to visualize alignment of assembled WT and △*chaB* genomes. B) Zoom-in of loci where *chaB* gene is localized. WT and △*chaB* are not properly aligned reflecting the insertion of chloramphenicol in place of *chaB* in the mutant strain.
